# PERSPECTIVES ON SENSORIZATION IN THE NURSING HOME SETTING AMONG LEADERS, STAFF, RESIDENTS, AND FAMILY MEMBERS

**DOI:** 10.1093/geroni/igae098.0070

**Published:** 2024-12-31

**Authors:** Sarah Holmes, Kelly Doran, Zainab Balogun, Yoon Chung Kim, Lewis Davis, Luke Zimmerman, Roberto Yus, Tera Reynolds

**Affiliations:** University of Maryland School of Nursing, Baltimore, Maryland, United States; University of Maryland Baltimore, Baltimore, Maryland, United States; University of Maryland Baltimore County, Baltimore, Maryland, United States; University of Maryland Baltimore, Baltimore, Maryland, United States; University of Maryland Baltimore County, Baltimore, Maryland, United States; University of Maryland Baltimore County, Baltimore, Maryland, United States; University of Maryland Baltimore County, Baltimore, Maryland, United States; University of Maryland Baltimore County, Baltimore, Maryland, United States

## Abstract

Sensorization, the process of adding smart devices (e.g., wearable or motion sensors) to spaces that can sense the environment and connect to the Internet, has numerous benefits such as improving patient outcomes, reducing clinical errors, and measuring provider stress to offer real-time interventions. However, decisions about which sensors to use can involve trade-offs including cost, accuracy, usability, and privacy. We sought to describe stakeholder perspectives toward sensorization in the nursing home. Guided by community-based participatory principles, this descriptive study included place-based observations of the nursing home environment, structured surveys to identify a priority goal that can be addressed with sensors, and qualitative interviews. The purposive sample of 25 participants included nursing home leaders (n=2), care staff (n=5), residents (n=10) and family members (n=8). Surveys were analyzed using descriptive statistics. The top priority goal identified was staff stress reduction and additional goals were resident fall prevention and staff workflow and efficiency. Sensorization plans were developed and tailored to address this goal, and interviews were conducted with participants (n=25) to understand their perceptions of the plans. Interviews were audio recorded, transcribed, and thematically analyzed in NVivo12. Themes were identified in four topic areas: 1) information types collected by sensors; 2) data sharing privacy concerns; 3) sensor location and usability; and 4) implementation feasibility. Findings shed light on the unique perspectives of the stakeholders on how they view and value the use of sensors which will inform additional research on sensorization in nursing homes to overcome implementation barriers using a systematic, participatory process.

